# Diagnostic challenges: low-grade adenosarcoma on deep endometriosis

**DOI:** 10.1186/s12905-019-0815-1

**Published:** 2019-10-26

**Authors:** Jose Carlos Vilches Jimenez, Emilia Villegas Muñoz, Iván González Poveda, David Santos Lorente, Belinda Sanchez Pérez, Jesús S. Jimenez Lopez

**Affiliations:** 1 From the Department of Obstetrics and Gynecology, Hospital Regional Universitario Malaga, Malaga, Spain; 2 From the Department of General Surgery, Hospital Regional Universitario Malaga, Malaga, Spain

**Keywords:** Endometriosis, Tumor, Adenosarcoma, Extrauterine, Laparoscopy

## Abstract

**Background:**

Müllerian adenosarcoma is a rare malignancy. These tumors occur mainly in the uterus, but also in extrauterine locations, usually related to endometriosis. Because of their rarity, there is limited data on optimal management strategies.

**Case presentation:**

We present a 44-year-old woman with a history of endometriosis who consults for chronic pelvic pain. In the imaging tests, a heterogeneous mass is observed that impresses endometriosis, encompassing the uterus and left appendage. Surgery is performed by finding an extrauterine adenosarcoma that affected the uterus, ovary and bladder wall.

**Conclusion:**

This is a rare case but should be considered in a patient with atypical clinical characteristics or preoperative pathology, so we show the diagnostic and therapeutic strategies carried out for the resolution of the case.

## Background

Endometriosis is a common condition among women of reproductive age. Although considered a benign disease, malignant transformation is possible. The incidence is higher in ovarian lesions (6–8%) and rare in extraovarian endometriosis (1%) [1]. The most common histology is endometriode adenocarcinoma and clear cell carcinoma. Uterine adenosarcomas make up 5% of uterine sarcomas [2]. Müllerian adenosarcomas are rare malignant tumors, unusual in young women. Their origin is usually the uterus, but they can also arise in extrauterine places (ovary, vagina, fallopian tube, broad and round ligaments, Douglas’ sac fundus, intestinal serosa and liver) generally related to endometriosis [2, 3]. They are usually low-grade tumors, containing benign epithelium with malignant mesenchymal components [4]. Prognosis depends on stage, depth of tumor invasion, grade, mitotic index, and the presence of heterologous elements. Sarcomatous overgrowth (defined as the presence of pure sarcoma occupying at least 25% of the tumor) is associated with poorer prognosis, recurrence and metastasis [5].

In this clinical case, we present a low-grade adenocarcinoma that arising in deep endometriosis. The patient was informed and gave her written consent to the elaboration and publication of this review. Similarly, the ethics committee of our centre gave its approval.

## Case presentation

44 year old patient, nulligest, with a history of deep endometriosis. Intervention of appendectomy, tonsillectomy and Toilette via laparoscopic, remitted for severe abdominal pain.

Followed for 3 years, in another center. Current treatment with gestagen, dienogest, comes to our center for severe abdominal pain requiring admission. Asked about menstrual symptoms, the patient reports spotting and irregular bleeding.

Provides additional evidence; transvaginal ultrasound: 3 cm intramural myoma, left ovary (39 × 28 mm) 18 mm cyst of endometriosis appearance; adjacent to ovary tubular heterogeneous formation of 54 × 23 mm (tube / hydrosalpinx). Blood test on admission: hemogram, coagulation and biochemistry were normal, CRP 5.2 and BHCG negative. Elevated tumor markers CA 125253.7.

She is discharged for clinical improvement and a full outpatient study is completed.

**Fig. 1:**
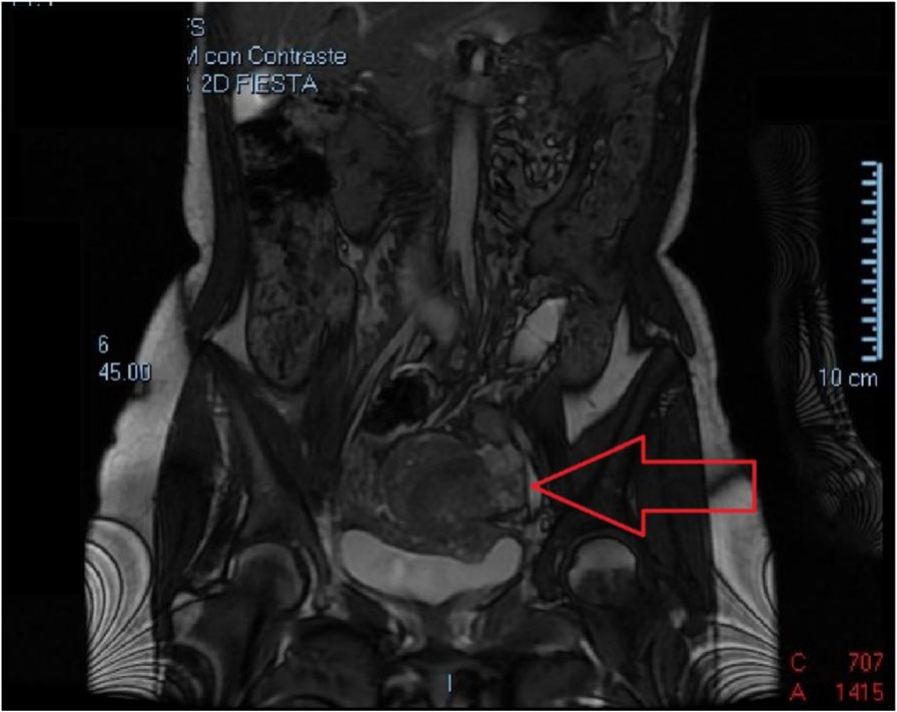
Heterogeneous lesion adhered to the left lateral side of the uterus

Rectosigmoidoscopy: without pathological findings, CT and MRI scan present in contact with the lateral wall of the uterus and without separation, an imprecise edge lesion of approximately 10 cm that extends throughout the left hemipelvis, inside which there are cystic areas, solids and several hemorrhagic foci, this lesion includes the left annex. To rule out Carcinoma of ovary as first diagnosis. (Fig. 1).

**Fig. 2:**
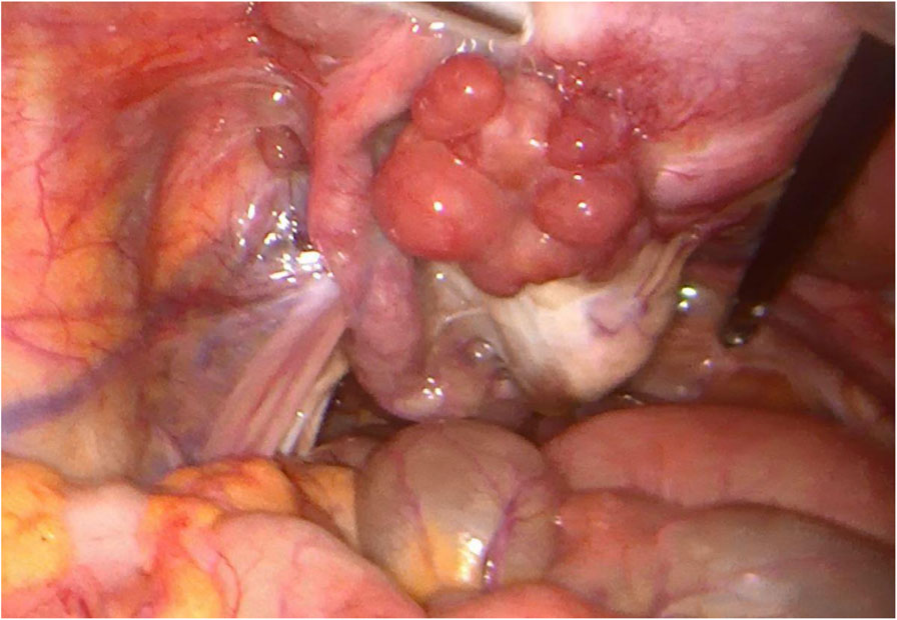
Mass dependent on left attachment

**Fig. 3:**
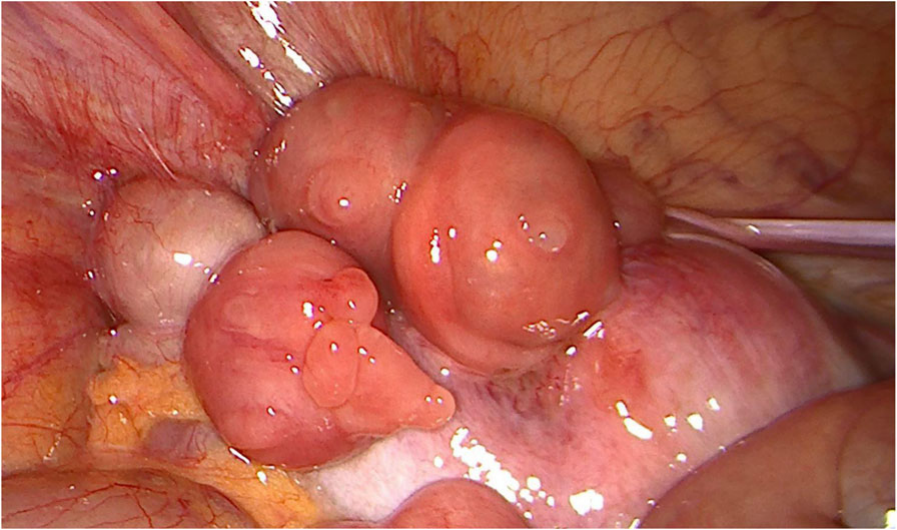
Anterior uterine mass

In the course of the surgical approach the following intraoperative findings were found: No ascites, no peritoneal neoplastic disease, multiple peritoneal endometriosis implants. Mass of 40 mm depending on the left annex, with multiple excrescences, infiltrative in left iliac fossa, enlarged uterus and adenomyotic appearance. Anterior uterine mass with loss of cleavage plane with posterior face of bladder, macroscopically normal right appendages, no involvement of Douglas or bowel loops. (Figs. 2 and 3).

Three intraoperative biopsies were performed (biopsy of left adnexal lesion, bladder tissue and left para-anexial lesion) due to the unusual nature of the lesions and their infiltration, all reported as endometriosis. Double J placement is performed and then total hysterectomy with double adnexectomy + partial bladder resection at the posterior face level + resection of endometriosis implant in left iliac fossa is performed.

Postoperative evolution favorable, the patient receiving discharge postoperatively on the 4th postoperative day.

The result of the surgical piece was: a uterus with an inactive endometrium, extensive adenomyosis, a 3 cm intramural myoma and low-grade adenosarcoma. Left ovary with a fibroid and low-grade adenosarcoma. Fragment of bladder wall with low-grade adenosarcoma infiltration. Adenosarcoma samples with estrogen receptor positive 100%, progesterone receptor positive100% and CD10 positive .

Differential diagnosis with polypoid endometriosis was proposed, despite the stromal cellularity with a pattern of low-grade stromal sarcoma with mild and focally moderate cytological atypia as well as the histological pattern (areas with polypoid projections in glandular lights) and the behavior indicate without a doubt that it is a low-grade adenosarcoma.

The oncology committee decides on treatment with hormone therapy, with Letrozole being the standard. Control at 3 with imaging test (CT) without observing alterations or new lesions. Control at 6 months, the patient being asymptomatic and with good quality of life.

## Discussion and Conclusion

A mullerian adenosarcoma is an exceptional tumor characterized by a generally low malignant stromal component and a benign glandular epithelial component [4, 6]. These tumors occur primarily in the uterus, but also in extra-uterine locations [2, 3] Extra-uterine tumors are thought to arise from deposits of endometriosis. In our case, we present an adenosarcoma with a low degree of extrauterine localization and with involvement of the uterus, ovary and bladder wall.

The risk of developing adenosarcoma increases in post-menopause age, with the average age at diagnosis being between 50 and 60 years. However, in the case of extra-uterine müllerian adenosarcomas, presentation in younger women is common, with an average age at diagnosis of 44 years. These tumours present a more aggressive behaviour, probably due to their location, which allows them to spread through the peritoneal cavity [7].

With respect to treatment, surgery is usually sufficient and curative in mullerian uterine adenosarcomas, with a recurrence rate of approximately 25% of cases. On the other hand, in the case of extra-uterine adenosarcomas the recurrence rate increases above 50% of patients and mortality rates are approximately of 35% [7].

The pathogenesis of the tumor is unclear. However, the association between exogenous hormone therapy and the development of malignant tumours in endometriosis is well known, and several studies have suggested the possibility of a neoplastic transformation of endometriosis sites [1].

A large Stern review of endometriosis-associated neoplasms showed that the most common malignancies were endometriode adenocarcinoma and clear cell carcinoma, with adenosarcoma mülleriano being much less common [2, 8].

This case meets the Sampson [9] criteria for malignant transformation of endometriosis, which include the following: endometriosis sites were found very close to the malignancy, the histological appearance was compatible with an endometrial origin, and no other possible primary tumor was observed.

Extrauterine tumors are clearly less common and are located mainly in the ovary, vagina, fallopian tube, broad and round ligaments, bottom of the Douglas sac, intestinal serosa and liver [2, 3].

The low incidence of Mullerian adenosarcomas complicates the development of well-defined and consensual action protocols. The main therapeutic weapon continues to be radical surgery and a similar approach to that used in endometrial carcinoma is usually recommended. The extent of muscle invasion and sarcomatous growth have a great influence when considering adjuvant chemotherapy. Adjuvancy is also used in the extra-uterine müllerian adenosarcoma although its usefulness is still debatable [7].

The most important negative prognostic factors are the location of the lesion, the degree of myometrial invasion and sarcomatous overgrowth [5]. In contrast, a favorable prognostic factor in patients with adenosarcoma is the presence of endometriosis [8].

Our case shows an association with endometriosis, which supports the hypothesis that an extra-uterine müllerian adenosarcoma can be transformed from this disease. The action of exogenous and endogenous oestrogens is currently being studied due to their possible relationship with the transformation process. It is also thought that the titles of the CA 125 tumour marker, that are well related to the clinical course of endometriosis, may become a predictor of sarcomatous overgrowth [7].

In conclusion, an extra-uterine müllerian adenosarcoma is rare, but should be considered in a patient with atypical clinical features or preoperative pathology, especially in a patient with a history of endometriosis. More research is needed to establish the pathogenesis of extra-uterine adenosarcoma and the therapeutic and diagnostic guidelines.

## Data Availability

Data sharing is not applicable to this article as no datasets were generated or analysed during the current study.

## Abbreviations

BHCG: Human chorionic gonadotropin
CRP: C-Reactive Protein
CT: Computed tomography
MRI: Magnetic resonance imaging
